# Supplementary material for: A Critical View of the Application of the APEX Software (Aqueous Photochemistry of Environmentally-Occurring Xenobiotics) to Predict Photoreaction Kinetics in Surface Freshwaters
Source: Molecules. 2019 Dec 18;25(1):9. doi: 10.3390/molecules25010009 (PMC7017383; doi:10.3390/molecules25010009)
Supplement: Supplementary file 1 [file molecules-25-00009-s001.zip › 3.2.4_gcc-4.4.0.zip!/3.2.4_gcc-4.4.0/3.2.4_gcc-4.4.0/doc/octave/html/interpreter/Functions-of-One-Variable.html]

Functions of One Variable - Untitled


Next: Functions of Multiple Variables,
Up: Numerical Integration


---

### 22.1 Functions of One Variable

Octave supports three different algorithms for computing the integral
of a function f over the interval from a to b.
These are

`quad`: Numerical integration based on Gaussian quadrature. `quadl`: Numerical integration using an adaptive Lobatto rule. `quadgk`: Numerical integration using an adaptive Gauss-Konrod rule. `quadv`: Numerical integration using an adaptive vectorized Simpson's rule. `trapz`: Numerical integration using the trapezoidal method.

Besides these functions Octave also allows you to perform cumulative
numerical integration using the trapezoidal method through the
`cumtrapz` function.

— Loadable Function: [v, ier, nfun, err] = **quad** (f, a, b, tol, sing)  
> Integrate a nonlinear function of one variable using Quadpack.
> The first argument is the name of the function, the function handle or
> the inline function to call to compute the value of the integrand. It
> must have the form
>
> ```
>           y = f (x)
> ```
>
> where y and x are scalars.
>
> The second and third arguments are limits of integration. Either or
> both may be infinite.
>
> The optional argument tol is a vector that specifies the desired
> accuracy of the result. The first element of the vector is the desired
> absolute tolerance, and the second element is the desired relative
> tolerance. To choose a relative test only, set the absolute
> tolerance to zero. To choose an absolute test only, set the relative
> tolerance to zero.
>
> The optional argument sing is a vector of values at which the
> integrand is known to be singular.
>
> The result of the integration is returned in v and ier
> contains an integer error code (0 indicates a successful integration).
> The value of nfun indicates how many function evaluations were
> required, and err contains an estimate of the error in the
> solution.
>
> You can use the function `quad_options` to set optional
> parameters for `quad`.
>
> It should be noted that since `quad` is written in Fortran it
> cannot be called recursively.

— Loadable Function: **quad\_options** (opt, val)  
> When called with two arguments, this function
> allows you set options parameters for the function `quad`.
> Given one argument, `quad_options` returns the value of the
> corresponding option. If no arguments are supplied, the names of all
> the available options and their current values are displayed.
>
> Options include
>
> `"absolute tolerance"`: Absolute tolerance; may be zero for pure relative error test. `"relative tolerance"`: Nonnegative relative tolerance. If the absolute tolerance is zero, the relative tolerance must be greater than or equal to `max (50*eps, 0.5e-28)`. `"single precision absolute tolerance"`: Absolute tolerance for single precision; may be zero for pure relative error test. `"single precision relative tolerance"`: Nonnegative relative tolerance for single precision. If the absolute tolerance is zero, the relative tolerance must be greater than or equal to `max (50*eps, 0.5e-28)`.

Here is an example of using `quad` to integrate the function

```
       f(x) = x * sin (1/x) * sqrt (abs (1 - x))
```

from x = 0 to x = 3.

This is a fairly difficult integration (plot the function over the range
of integration to see why).

The first step is to define the function:

```
     function y = f (x)
       y = x .* sin (1 ./ x) .* sqrt (abs (1 - x));
     endfunction
```

Note the use of the `dot' forms of the operators. This is not necessary
for the call to `quad`, but it makes it much easier to generate a
set of points for plotting (because it makes it possible to call the
function with a vector argument to produce a vector result).

Then we simply call quad:

```
     [v, ier, nfun, err] = quad ("f", 0, 3)
          => 1.9819
          => 1
          => 5061
          => 1.1522e-07
```

Although `quad` returns a nonzero value for ier, the result
is reasonably accurate (to see why, examine what happens to the result
if you move the lower bound to 0.1, then 0.01, then 0.001, etc.).

— Function File: q = **quadl** (f, a, b)  
— Function File: q = **quadl** (f, a, b, tol)  
— Function File: q = **quadl** (f, a, b, tol, trace)  
— Function File: q = **quadl** (f, a, b, tol, trace, p1, p2, ...)  
> Numerically evaluate integral using adaptive Lobatto rule.
> `quadl (`f`,` a`,` b`)` approximates the integral of
> f`(`x`)` to machine precision. f is either a
> function handle, inline function or string containing the name of
> the function to evaluate. The function f must return a vector
> of output values if given a vector of input values.
>
> If defined, tol defines the relative tolerance to which to
> which to integrate f`(`x`)`. While if trace is
> defined, displays the left end point of the current interval, the
> interval length, and the partial integral.
>
> Additional arguments p1, etc., are passed directly to f.
> To use default values for tol and trace, one may pass
> empty matrices.
>
> Reference: W. Gander and W. Gautschi, 'Adaptive Quadrature -
> Revisited', BIT Vol. 40, No. 1, March 2000, pp. 84–101.
> http://www.inf.ethz.ch/personal/gander/

— Function File: **quadgk** (f, a, b, abstol, trace)  
— Function File: **quadgk** (f, a, b, prop, val, ...)  
— Function File: [q, err] = **quadgk** (...)  
> Numerically evaluate integral using adaptive Gauss-Konrod quadrature.
> The formulation is based on a proposal by L.F. Shampine,
> "Vectorized adaptive quadrature in matlab", Journal of
> Computational and Applied Mathematics, pp131-140, Vol 211, Issue 2,
> Feb 2008 where all function evaluations at an iteration are
> calculated with a single call to f. Therefore the function
> f must be of the form f `(`x`)` and accept
> vector values of x and return a vector of the same length
> representing the function evaluations at the given values of x.
> The function f can be defined in terms of a function handle,
> inline function or string.
>
> The bounds of the quadrature `[`a`,` b`]` can be finite
> or infinite and contain weak end singularities. Variable
> transformation will be used to treat infinite intervals and weaken
> the singularities. For example
>
> ```
>           quadgk(@(x) 1 ./ (sqrt (x) .* (x + 1)), 0, Inf)
> ```
>
> Note that the formulation of the integrand uses the
> element-by-element operator `./` and all user functions to
> `quadgk` should do the same.
>
> The absolute tolerance can be passed as a fourth argument in a manner
> compatible with `quadv`. Equally the user can request that
> information on the convergence can be printed is the fifth argument
> is logically true.
>
> Alternatively, certain properties of `quadgk` can be passed as
> pairs prop`,` val. Valid properties are
>
> `AbsTol`: Defines the absolute error tolerance for the quadrature. The default absolute tolerance is 1e-10. `RelTol`: Defines the relative error tolerance for the quadrature. The default relative tolerance is 1e-5. `MaxIntervalCount`: `quadgk` initially subdivides the interval on which to perform the quadrature into 10 intervals. Sub-intervals that have an unacceptable error are sub-divided and re-evaluated. If the number of sub-intervals exceeds at any point 650 sub-intervals then a poor convergence is signaled and the current estimate of the integral is returned. The property 'MaxIntervalCount' can be used to alter the number of sub-intervals that can exist before exiting. `WayPoints`: If there exists discontinuities in the first derivative of the function to integrate, then these can be flagged with the `"WayPoints"` property. This forces the ends of a sub-interval to fall on the breakpoints of the function and can result in significantly improved estimation of the error in the integral, faster computation or both. For example, ``` quadgk (@(x) abs (1 - x .^ 2), 0, 2, 'Waypoints', 1) ``` signals the breakpoint in the integrand at x `= 1`. `Trace`: If logically true, then `quadgk` prints information on the convergence of the quadrature at each iteration.
>
> If any of a, b or waypoints is complex, then the
> quadrature is treated as a contour integral along a piecewise
> continuous path defined by the above. In this case the integral is
> assumed to have no edge singularities. For example
>
> ```
>           quadgk (@(z) log (z), 1+1i, 1+1i, "WayPoints",
>                   [1-1i, -1,-1i, -1+1i])
> ```
>
> integrates `log (z)` along the square defined by `[1+1i,
> 1-1i, -1-1i, -1+1i]`
>
> If two output arguments are requested, then err returns the
> approximate bounds on the error in the integral `abs (`q `-` i`)`, where i is the exact value of the integral.
>
>
>
> **See also:** triplequad, dblquad, quad, quadl, quadv, trapz.

— Function File: q = **quadv** (f, a, b)  
— Function File: q = **quadl** (f, a, b, tol)  
— Function File: q = **quadl** (f, a, b, tol, trace)  
— Function File: q = **quadl** (f, a, b, tol, trace, p1, p2, ...)  
— Function File: [q, fcnt] = **quadl** (...)  
> Numerically evaluate integral using adaptive Simpson's rule.
> `quadv (`f`,` a`,` b`)` approximates the integral of
> f`(`x`)` to the default absolute tolerance of `1e-6`.
> f is either a function handle, inline function or string
> containing the name of the function to evaluate. The function f
> must accept a string, and can return a vector representing the
> approximation to n different sub-functions.
>
> If defined, tol defines the absolute tolerance to which to
> which to integrate each sub-interval of f`(`x`)`.
> While if trace is defined, displays the left end point of the
> current interval, the interval length, and the partial integral.
>
> Additional arguments p1, etc., are passed directly to f.
> To use default values for tol and trace, one may pass
> empty matrices.
>
>
>
> **See also:** triplequad, dblquad, quad, quadl, quadgk, trapz.

— Function File: z = **trapz** (y)  
— Function File: z = **trapz** (x, y)  
— Function File: z = **trapz** (..., dim)  
> Numerical integration using trapezoidal method. `trapz
> (`y`)` computes the integral of the y along the first
> non-singleton dimension. If the argument x is omitted a
> equally spaced vector is assumed. `trapz (`x`,` y`)`
> evaluates the integral with respect to x.
>
>
>
> **See also:** cumtrapz.

— Function File: z = **cumtrapz** (y)  
— Function File: z = **cumtrapz** (x, y)  
— Function File: z = **cumtrapz** (..., dim)  
> Cumulative numerical integration using trapezoidal method.
> `cumtrapz (`y`)` computes the cumulative integral of the
> y along the first non-singleton dimension. If the argument
> x is omitted a equally spaced vector is assumed. `cumtrapz
> (`x`,` y`)` evaluates the cumulative integral with respect
> to x.
>
>
>
> **See also:** trapz, cumsum.
